# Targeted inhibition of Hsp90 by ganetespib is effective across a broad spectrum of breast cancer subtypes

**DOI:** 10.1007/s10637-013-9971-6

**Published:** 2013-05-18

**Authors:** Julie C. Friedland, Donald L. Smith, Jim Sang, Jaime Acquaviva, Suqin He, Chaohua Zhang, David A. Proia

**Affiliations:** Synta Pharmaceuticals Corp., 125 Hartwell Avenue, Lexington, MA 02421 USA

**Keywords:** Hsp90 inhibition, Ganetespib, Breast cancer, Cancer therapy

## Abstract

Heat shock protein 90 (Hsp90) is a molecular chaperone essential for the stability and function of multiple cellular client proteins, a number of which have been implicated in the pathogenesis of breast cancer. Here we undertook a comprehensive evaluation of the activity of ganetespib, a selective Hsp90 inhibitor, in this malignancy. With low nanomolar potency, ganetespib reduced cell viability in a panel of hormone receptor-positive, HER2-overexpressing, triple-negative and inflammatory breast cancer cell lines in vitro. Ganetespib treatment induced a rapid and sustained destabilization of multiple client proteins and oncogenic signaling pathways and even brief exposure was sufficient to induce and maintain suppression of HER2 levels in cells driven by this receptor. Indeed, HER2-overexpressing BT-474 cells were comparatively more sensitive to ganetespib than the dual HER2/EGFR tyrosine kinase inhibitor lapatinib in three-dimensional culture. Ganetespib exposure caused pleiotropic effects in the inflammatory breast cancer line SUM149, including receptor tyrosine kinases, MAPK, AKT and mTOR signaling, transcription factors and proteins involved in cell cycle, stress and apoptotic regulation, as well as providing combinatorial benefit with lapatinib in these cells. This multimodal activity translated to potent antitumor efficacy in vivo, suppressing tumor growth in MCF-7 and MDA-MB-231 xenografts and inducing tumor regression in the BT-474 model. Thus, ganetespib potently inhibits Hsp90 leading to the degradation of multiple clinically-validated oncogenic client proteins in breast cancer cells, encompassing the broad spectrum of molecularly-defined subtypes. This preclinical activity profile suggests that ganetespib may offer considerable promise as a new therapeutic candidate for patients with advanced breast cancers.

**Electronic supplementary material:**

The online version of this article (doi:10.1007/s10637-013-9971-6) contains supplementary material, which is available to authorized users.

## Introduction

Breast cancer remains the most commonly diagnosed female malignancy and principal cause of cancer-related mortality in women worldwide [1]. Tumors of the breast show a remarkable degree of cellular and molecular heterogeneity such that breast cancer is no longer considered a single disease with variable morphology, but rather a collection of distinct neoplastic disorders [2], each associated with their own pathological features and clinical outcome. Gene expression profiling has resulted in the classification of human breast cancer into at least four subtypes based on discrete molecular signatures [3–6]. These include the luminal A and luminal B subtypes, which are positive for estrogen and progesterone receptors (ER and PR), human epidermal growth factor receptor 2 (HER2)-positive, and basal-like. HER2-positive breast cancer is characterized by amplification of the *HER2* oncogene and overexpression of the receptor while basal-like tumors express specific genes characteristic of basal epithelial/myoepithelial cells. Triple negative breast cancers (TNBC), an orphan grouping of tumors which lack expression of ER, PR and HER2, primarily fall into the basal-like subtype, although the two definitions are not synonymous [7–9].

This stratification of breast cancer also carries prognostic significance in terms of clinical behavior and response to therapy. In general, poorer outcomes are seen for the two hormone receptor-negative subtypes compared to the luminal subgroups. However, even though both luminal A and luminal B breast cancers are ER-positive, luminal B cancers have a considerably worse prognosis, with overall survival in untreated tumors similar to that of the HER2-positive and basal types [2]. Moreover, luminal B tumors display a higher relative resistance to endocrine therapy, such as with the selective ER modulator tamoxifen, than luminal-A tumors [2, 10]. HER2-positive breast cancer is an aggressive disease, with HER2 overexpression representing a significant negative predictor of both overall survival and time to relapse [11]. Fortunately the prognosis for HER2-positive breast cancer patients has significantly improved since the introduction of selective HER2-targeted agents (such as trastuzumab and lapatinib) as first-line treatments [12]. In contrast, due to an absence of molecular targets, chemotherapy is the only therapeutic option in the adjuvant or metastatic setting for TNBC tumors [8]. Consequently these cancers remain high risk with particularly unfavorable prognoses [9, 13].

Heat shock protein 90 (Hsp90) is a molecular chaperone that plays an indispensable role in normal cellular homeostasis by regulating the folding, stability, and function of its target substrates, termed “client” proteins [14]. During tumorigenesis, the chaperoning activity of Hsp90 may become co-opted by cancer cells, in turn conferring aberrant proliferative, survival, angiogenic and/or metastatic potential [15, 16]. Indeed, a number of sensitive Hsp90 clients have been implicated in the pathogenesis of breast cancer, including steroid hormone receptors (ER and PR), receptor tyrosine kinases (HER2, epidermal growth factor receptor (EGFR)) and intermediates of oncogenic signaling cascades (AKT and RAF1) [17]. Inhibition of Hsp90 activity causes client proteins to adopt aberrant conformations, triggering ubiquitination and proteasomal degradation. In this regard, Hsp90 blockade provides a means to simultaneously target multiple oncogenic signaling pathways [18, 19] and Hsp90 has therefore become an attractive molecular target for the development of new anticancer agents [20, 21]. There is considerable preclinical evidence to support the potential utility of Hsp90 inhibitors in breast cancer [22–28]. Further, clinical benefit has been observed following the addition of the first-generation Hsp90 inhibitor tanespimycin (17-AAG) to trastuzumab in HER2-positive metastatic breast cancer [29], thus providing important proof-of-concept for the rational design of combinatorial strategies to improve patient outcomes. Despite this progress, however, no Hsp90 inhibitors have yet been approved for the treatment of any human cancer.

Ganetespib is a small molecule triazolone inhibitor of Hsp90 with an antitumor activity, potency and safety profile distinct from, and superior to, other first- and second-generation inhibitors [30]. In preclinical studies ganetespib showed robust activity against a range of cancer models including lung, prostate, and leukemia [31–35]. As predicted by these findings, a maturing clinical profile has revealed evidence of therapeutic activity in human tumors, particularly in non-small cell lung cancer where ganetespib has shown promising single-agent efficacy in molecularly defined subsets of that disease [36]. Here we have undertaken a comprehensive evaluation of ganetespib activity in breast cancer cell lines that encompass both luminal and basal histologies, including hormone receptor-positive, HER2-positive and TNBC subtypes, as well as inflammatory breast cancer (IBC). The results suggest that ganetespib offers considerable promise as an alternative, and potentially complementary, therapeutic strategy to target breast cancer. In light of these considerations a global Phase II study evaluating ganetespib as a front-line treatment for metastatic breast cancer (ENCHANT Trial, NCT01677455) has recently been initiated.

## Materials and methods

### Cell lines, antibodies and reagents

The MCF-7, T47D, BT-474, MDA-MB-231 and Sk-BR3 cell lines were obtained from the American Type Culture Collection (ATCC, Manassas, VA, USA) and maintained at 37 °C in 5 % (v/v) CO_2_ using culture medium recommended by the supplier. OCUB-M cells were obtained from Riken/Wako (Osaka, Japan). SUM149 cells were purchased from Asterand (Detroit, MI, USA) and were cultured in Ham’s F12 media supplemented with 5 % FBS, 1 ug/ml hydrocortisone, 5 ug/ml insulin and antibiotics. All primary antibodies were purchased from Cell Signaling Technology (CST, Beverly, MA, USA) with the exception of p-EGFR and p-HER2 (Invitrogen, Grand Island, NY, USA) and the ER and GAPDH antibodies (Santa Cruz Biotechnology Inc., Santa Cruz, CA). Ganetespib [3-(2,4-dihydroxy-5-isopropylphenyl)-4-(1-methyl-1H-1,2,4-triazol-5(4H)-one] was synthesized by Synta Pharmaceuticals Corp. Lapatinib was purchased from LC Laboratories (Woburn, MA, USA), and doxorubicin from Sigma-Aldrich (St. Louis, MO, USA).

### Cell viability assays

Cellular viability was assessed using the CellTiter-Glo Luminescent Cell Viability Assay (Promega, Madison, WI, USA) according to the manufacturer’s protocol. Breast cancer cell lines were seeded into 96-well plates based on optimal growth rates determined empirically for each line. Twenty-four hours after plating, cells were dosed with graded concentrations of drug for 72 h. CellTiter-Glo was added (50 % v/v) to the cells, and the plates incubated for 10 min prior to luminescent detection in a Victor 2 microplate reader (Perkin Elmer, Waltham, MA, USA). Data were normalized to percent of control and IC_50_ values were determined using XLFit software. Mammosphere cultures of BT-474 were produced as previously described [37] prior to addition of ganetespib or lapatanib at a final concentration of 41 nM and 124 nM for 72 h.

### Western blotting

Following in vitro assays, tumor cells were disrupted in lysis buffer (CST) on ice for 10 min. For the pharmacodynamic analysis, xenograft tumors (average volume ~210 mm^3^) were excised, cut in half, and flash frozen in liquid nitrogen. Each tumor fragment was lysed in 0.5 mL of lysis buffer using a FastPrep-24 homogenizer and Lysing Matrix A (MP Biomedicals, Solon, OH). Lysates were clarified by centrifugation and equal amounts of proteins resolved by SDS-PAGE before transfer to nitrocellulose membranes (Bio-Rad, Hercules, CA, USA). Membranes were blocked with StartingBlock T20 blocking buffer (Thermo Scientific, Cambridge, MA, USA) and immunoblotted with the indicated antibodies. Antibody-antigen complexes were visualized using an Odyssey system (LI-COR, Lincoln, NE, USA).

### In-Cell Western assay

BT-474 cells were seeded in 96-well plates and incubated for 24 h prior to the addition of vehicle or ganetespib to the culture. Ganetespib was added to the cells using 3 fold serial dilutions and incubated for the indicated time periods ranging from 5 min to 72 h. Growth media containing drug was aspirated off, cells were washed twice and incubated in growth medium for a total of 72 h. Cells were then fixed in 4 % paraformaldehyde for 20 min and washed three times in 0.1 % Triton X-100. After blocking for 1.5 h in Odyssey Blocking Buffer (LI-COR, Lincoln, NE, USA), cells were incubated with primary HER2 antibody overnight at 4 °C. Wells were washed three times with 0.1 % Tween-20 and incubated in secondary antibody (LI-COR, Lincoln, NE, USA) and DRAQ5 (CST, Beverly, MA, USA) for 1 h. Following 3 final washes with 0.1 % Tween-20, antibody-antigen complexes were visualized using the Odyssey system.

### In vivo breast tumor models

Female CB-17/SCID or CB-17/NOD.SCID mice (Charles River Laboratories, Wilmington, MA) at 7–12 weeks of age were maintained in a pathogen-free environment and all in vivo procedures were approved by the Synta Pharmaceuticals Corp. Institutional Animal Care and Use Committee. BT-474 (10 × 10^6^) and MDA-MB-231 (1 × 10^5^) cells were subcutaneously implanted into SCID mice and MCF-7 cells (5 × 10^6^) into NOD SCID animals. Mice bearing established tumors (150–250 mm^3^) were randomized into treatment groups of 8 and dosed with vehicle or ganetespib (i.v.) formulated in DRD (10 % DMSO, 18 % Cremophor RH 40, 3.6 % dextrose), using the schedules indicated. For the pharmacodynamic analysis, MCF-7 xenograft bearing mice were randomized into groups of 3 and administered a single i.v. injection of 125 mg/kg ganetespib and animals sacrificed 24, 72 and 96 h later. Vehicle-treated animals were sacrificed at the 24 h time point. Tumor volumes (V) were calculated by caliper measurements of the width (W), length (L) and thickness (T) of each tumor using the formula: V = 0.5236(LWT). Tumor growth inhibition was determined from the change in average tumor volumes of each treated group relative to the vehicle-treated, or itself in the case of tumor regression.

### Reverse phase protein array

SUM149 cells were treated with DMSO (control) or ganetespib (250 nM) for 24 h. Lysates were then prepared as recommended by the Reverse Phase Protein Analysis Core Facility at MD Anderson Cancer Center (Houston, TX, USA). Serial diluted lysates were arrayed on nitrocellulose-coated FAST slides (Whatman) and probed for a standard list of antibodies as previously described [38].

### Combinatorial drug effect analysis

For combinatorial analysis, cells were seeded in 96-well plates at a predetermined, optimum growth density and incubated at 37 °C, 5 % CO_2_ for 24 h prior to the addition of drug or vehicle to the culture. Drug combinations were applied at a non-constant ratio, using three 1.5 fold serial dilutions above and below the IC_50_ values for each compound. Cell viability was assessed 72 h after drug addition by Cell Titer-Glo and normalized to vehicle controls. For each combination study, the level of growth inhibition (fraction affected) is plotted relative to vehicle control. Data are presented as one relevant combination point and the corresponding single agent data for each cell line tested.

## Results

### Loss of ER/PR protein expression and viability in hormone receptor-positive breast cancer cells by ganetespib leads to robust antitumor efficacy in vivo

**Fig. 1:**
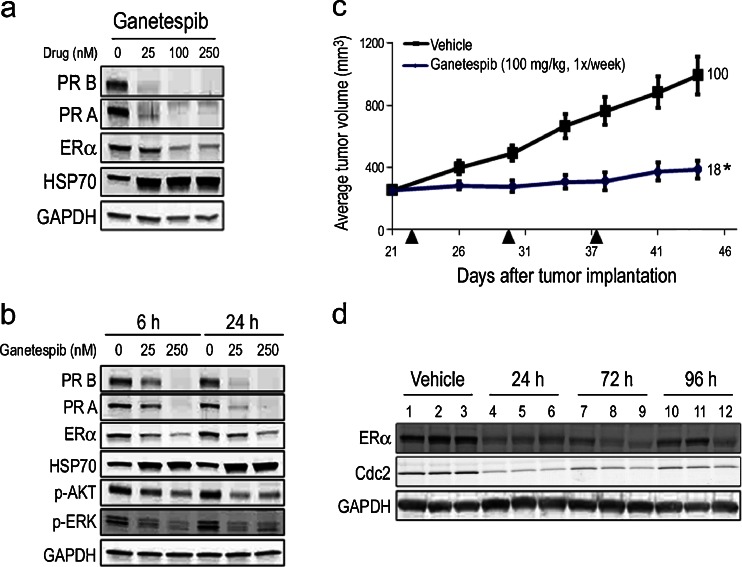
Ganetespib activity in hormone receptor-positive breast cancer cells in vitro and in vivo. **a** T47D cells were treated with increasing concentrations of ganetespib for 24 h. The levels of PR B, PR A, ERα and HSP70 were determined by immunoblotting. GAPDH was included as a loading control. **b** T47D cells were exposed to graded concentrations of ganetespib for 6 and 24 h as indicated. Cell lysates were immunoblotted using antibodies against PR B, PR A, ERα, HSP70, phosphorylated AKT (p-AKT), phosphorylated ERK (p-ERK), phosphorylated PDK1 (p-PDK1) and GAPDH as shown. **c** Mice bearing established MCF-7 xenografts (*n* = 8/group) were i.v. dosed with 100 mg/kg ganetespib once weekly over a 3 week cycle. % T/C values are indicated to the right of each growth curve and the error bars are the SEM. Ganetespib treatment significantly suppressed tumor growth (*, *p* < 0.05). **d** Pharmacodynamic analysis. Mice bearing MCF-7 tumors (*n* = 3/group) were treated with vehicle or a single bolus injection of ganetespib at 125 mg/kg for 24, 72 and 96 h. Tumors were resected and the levels of ERα, Cdc2 and GAPDH determined by immunoblotting

The majority of human breast cancers are luminal type and are predominantly comprised of tumors that express variable levels of estrogen receptor (ER) and/or progesterone receptor (PR). With low nanomolar potency, ganetespib reduced viability in two hormone receptor-positive cell lines, MCF-7 and T47D (Table 1), with IC_50_ values of 25 and 15 nM, respectively. Initially we investigated the effects of ganetespib exposure on receptor expression using T47D cells (Fig. 1a). Ganetespib treatment resulted in a potent and robust dose-dependent destabilization of both isoforms of PR (PR B and PR A) and ER. This was accompanied by increased HSP70 expression, which serves as a surrogate marker for Hsp90 inhibition. Next we examined the kinetics of steroid receptor loss and pathway modulation in T47D cells. As shown in Fig. 1b, 250 nM ganetespib treatment rapidly (within 6 h) induced complete destabilization of PR and maximal reductions in ER levels and these effects were sustained over a 24 h time course. Even a low 25 nM concentration of ganetespib resulted in measurable decreases in PR and ER expression levels by 6 h and these became more pronounced over time. Importantly, targeted degradation of these clients was accompanied by inactivation of downstream signaling intermediates including phosphorylated AKT and ERK (Fig. 1b).

**Table 1:** In vitro cytotoxicity of ganetespib and molecular profiling of breast cancer cell lines

Cell line	IC_50_ (nM)	Molecular sub-type	ER	PR	HER2 overexpression	Phenotype
MCF-7	25 ± 1.8	Luminal A	+	+	–	Hormone+
T47D	15 ± 3.2	Luminal A	+	+	–	Hormone+
BT-474	13 ± 1.7	Luminal B	+	+	+	HER2+
Sk-BR3	25 ± 5.3	Luminal	–	–	+	HER2+
SUM149	13 ± 1.6	Basal	–	–	–	IBC
MDA-MB-231	24 ± 1.6	Basal	–	–	–	TNBC
OCUB-M	39 ± 0.2	Basal	–	–	–	TNBC

Hormone +, hormone receptor expressing; HER2+, HER2 overexpressing; IBC, inflammatory breast cancer; TNBC, triple negative breast cancer. IC_50_ data are presented as means ± standard error of the mean

To examine whether these in vitro effects on cell signaling and viability translated to antitumor activity in vivo, we studied the efficacy of ganetespib treatment on the growth of hormone receptor-positive breast cancer xenografts. As shown in Fig. 1c, mice bearing MCF-7 xenografts that were treated on a weekly dosing regimen of ganetespib at 100 mg/kg exhibited a significant decrease in tumor volume compared to control animals (T/C value 18 %). In addition, the schedule was well tolerated, with no toxicity or changes in body weight observed over the 3 week period (data not shown). Next we determined whether this tumor response correlated with target modulation by performing pharmacodynamic analysis in additional MCF-7 tumor-bearing mice (Fig. 1d). Animals were treated with a single bolus injection of ganetespib at 125 mg/kg and the tumors harvested 24, 72 and 96 h after treatment. Expression of ER and the cell cycle regulator Cdc2 were significantly suppressed 24 h following ganetespib treatment. Importantly, these effects were sustained over time, as recovery of either was not underway before 96 h post-dosing.

### Potent and durable loss of client protein expression by ganetespib induces cell death and suppresses tumor growth in HER2-positive breast cancer models

**Fig. 2:**
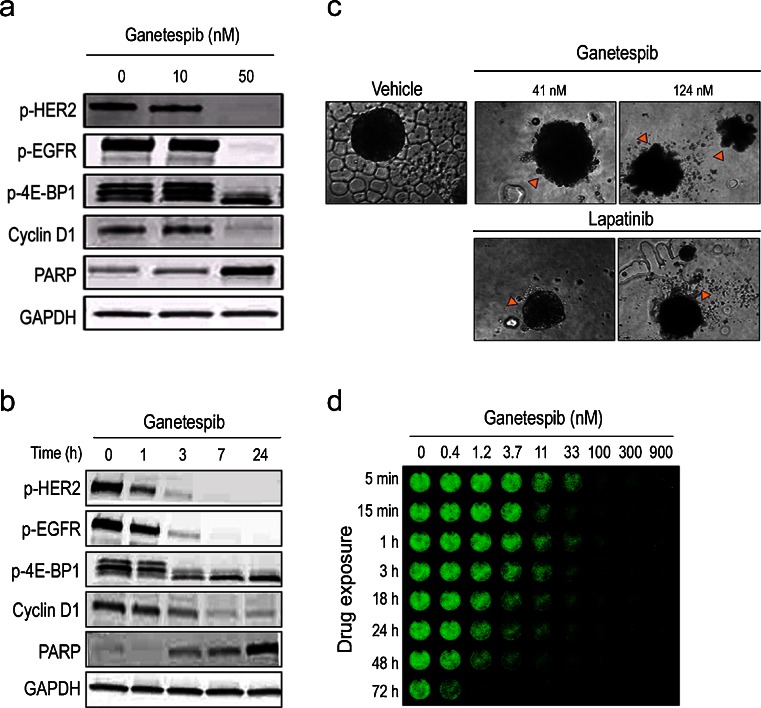
Potent and durable destabilization of HER2 inhibits oncogenic signaling and induces cell death in HER2+ breast cancer cells. **a** BT-474 cells treated with ganetespib at 0, 10 or 50 nM for 24 h. Cell lysates were immunoblotted using antibodies against phosphorylated HER2 (p-HER2), phosphorylated EGFR (p-EGFR), phosphorylated 4E-BP1 (p-4E-BP1), and Cyclin D1 as shown. Cleaved PARP expression was included as a marker of apoptosis and GAPDH as a loading control. **b** BT-474 cells treated with 250 nM ganetespib for 1, 3, 7 and 24 h. Cell lysates were immunoblotted using antibodies as listed above. **c** BT-474 cells were grown in 3D culture as mammospheres and treated with ganetespib or lapatanib at the indicated concentrations (41 or 124 nM) for 72 h. At both dose levels, extensive cell death was observed following ganetespib exposure; cytotoxicity was only seen at the higher dose for lapatinib. **d** In-cell Western (ICW) assay for HER2 expression. BT-474 cells were exposed to increasing concentrations of ganetespib (0–900 nM) for the indicated time periods, washed to remove drug and then grown in standard medium before fixation and analysis by ICW assay at the 72 h time point

HER2 overexpression is characteristic of 15–20 % of invasive breast cancers and, importantly, has been validated both as a prognostic factor and a predictive biomarker for HER2-targeted therapies [11, 39]. Ganetespib potently reduced viability in the HER2-positive breast cancer cell lines BT-474 and Sk-BR3, with IC_50_ values of 13 and 25 nM (Table 1). Ganetespib exposure resulted in the dose-dependent and complete degradation of active (phosphorylated) HER2 and EGFR receptors (Fig. 2a). Mammalian target of rapamycin (mTOR) signaling and cell cycle regulation were similarly affected by ganetespib treatment, as evidenced by reductions in phosphorylated 4E-BP1 protein levels and loss of Cyclin D1 expression, respectively. These changes were accompanied by an elevation of cleaved PARP expression, indicative of apoptotic induction (Fig. 2a). When the kinetics of client protein loss were examined, we found that treatment of BT-474 cells with 250 nM ganetespib resulted in a rapid (1–3 h) destabilization of HER2 and EGFR receptor proteins, with complete loss observed by 7 h and this was sustained over the 24 h time course (Fig. 2b). Perturbation of mTOR signaling, cell cycle disruption and apoptotic induction followed an identical time course (Fig. 2b). Similar results were observed in the Sk-BR3 cell line (data not shown).

BT-474 cells were also sensitive to the cytotoxic effects of ganetespib when grown as mammospheres in three-dimensional culture (Fig. 2c). Exposure to ganetespib for 72 h at either 41 nM or 124 nM resulted in extensive cell death and loss of spheroid structural integrity. Interestingly, when BT-474 mammospheres were treated with identical concentrations of the dual HER2/EGFR tyrosine kinase inhibitor lapatinib, cytotoxic effects were only observed at the higher dose (Fig. 2c). This finding suggests that ganetespib displays superior in vitro potency for inducing BT-474 cell death than lapatinib.

We have previously reported that brief exposure of lung cancer cell lines to ganetespib in vitro leads to persistent Hsp90 inhibitory activity and concomitant effects on viability [30, 32]. To explore the durable response property of ganetespib on HER2 oncoprotein expression, we exposed BT-474 cells to graded concentrations of drug for varying time periods and measured HER2 protein levels using an In-Cell Western assay. As expected, ganetespib treatment resulted in a time- and dose-dependent reduction in HER2 expression (Fig. 2d). Notably, even a 5 min exposure to ganetespib at concentrations ≥100 nM resulted in a sustained HER2 destabilization for 72 h. Thus transient, robust inhibition of Hsp90 by ganetespib was sufficient to induce and maintain suppression of HER2 protein levels in breast cancer cells driven by this receptor.

**Fig. 3:**
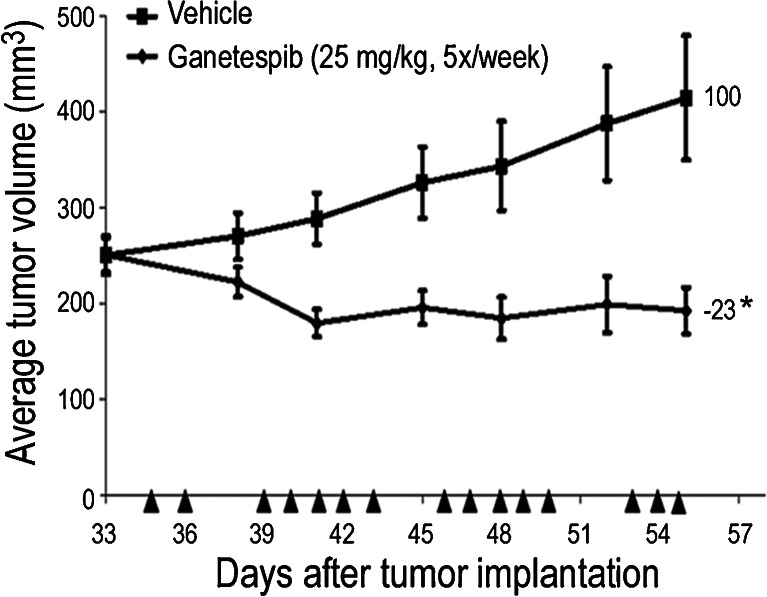
Ganetespib treatment induces tumor regression in HER2+ breast xenografts. Mice bearing established BT-474 xenografts (*n* = 8/group) were i.v. dosed with 25 mg/kg ganetespib 5×/week over a 3 week cycle. % T/C values are indicated to the right of each growth curve and the error bars are the SEM. Ganetespib treatment resulted in significant tumor regression (*, *p* < 0.05)

Next we evaluated the antitumor efficacy of ganetespib in HER2-positive breast tumors. Ganetespib administered at 25 mg/kg 5×/week over a 3 week cycle to mice bearing established BT-474 xenografts resulted in 23 % tumor regression (Fig 3). On this regimen some toxicity, as measured by body weight loss, was observed in 2/8 animals; thus this dose was determined to be the maximally tolerated dose (MTD).

### Ganetespib inhibits oncogenic signaling and tumor growth in triple-negative breast cancer cells

**Fig. 4:**
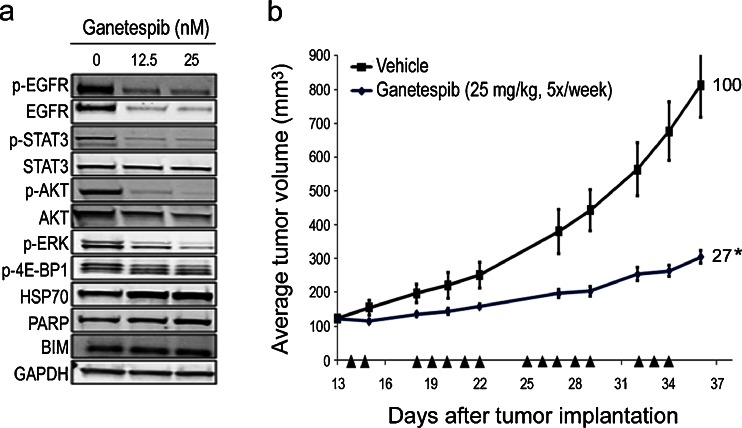
Ganetespib inhibits oncogenic signaling in TNBC cells and suppresses tumor growth. **a** MDA-MB-231 cells were exposed to low dose concentrations of ganetespib (12.5 and 25 nM) for 24 h. Cell lysates were immunoblotted using antibodies against phosphorylated EGFR (p-EGFR), total EGFR, phosphorylated STAT3 (p-STAT3), total STAT3, phosphorylated AKT (p-AKT), total AKT, phosphorylated 4E-BP1 (p-4E-BP1), HSP70, PARP and BIM as shown. GAPDH was included as a loading control. **b** Mice bearing established MDA-MB-231 xenografts (*n* = 8/group) were dosed with 25 mg/kg ganetespib (i.v.) 5×/week over a 3 week cycle. % T/C values are indicated to the right of each growth curve and the error bars are the SEM. Ganetespib treatment significantly suppressed tumor growth (*, *p* < 0.05)

TNBC represents a heterogeneous subset of basal-type tumors characterized by an absence of ER, PR and HER2 expression [8, 9]. Although the incidence of TNBC is only 10–20 %, these cancers show a disproportionate mortality for breast cancer patients [8]. Ganetespib was potently cytotoxic to the TNBC lines MDA-MB-231 and OCUB-M with IC_50_ values in the low nanomolar range (Table 1). Similar to the SUM149 cell line, MDA-MB-231 cells overexpress EGFR and this is a common feature of TNBC. Low-dose treatment of MDA-MB-231 cells with ganetespib abrogated both EGFR activity and expression of the receptor, as well as loss of downstream STAT3 activity (i.e. reduction in p-STAT3 levels) (Fig. 4a). This was accompanied by inactivation of AKT, ERK and mTOR signaling (p-4E-BP1) as well as a concomitant increase in the apoptotic markers PARP and BIM (Fig. 4a). By extension, we evaluated in vivo antitumor efficacy in mice bearing MDA-MB-231 xenografts that were administered 25 mg/kg ganetespib 5×/week over a 3 week cycle, which resulted in a 73 % suppression of tumor growth (Fig. 4b).

### Ganetespib inhibition of multiple oncogenic signaling pathways and targeted combinatorial activity in inflammatory breast cancer

Inflammatory breast cancer (IBC) is a rare, clinically distinct and aggressive form of locally advanced breast cancer [40]. Ganetespib was potently cytotoxic to the well-characterized IBC cell line SUM149, with an IC_50_ value of 13 nM (Table 1). Because the underlying biology of IBC remains poorly understood, we performed reverse phase array analysis to examine the profile of proteins in SUM149 cells whose expression was modulated following ganetespib treatment (Table 2). In addition to promoting targeted loss of client protein receptors such as EGFR and IGF-IR, ganetespib exposure selectively altered the expression of oncogenic signaling intermediates of both the MAPK (Src, Shc, C-RAF, JNK, MAPK) and mTOR signaling pathways (mTOR, S6, 4E-BP1, PRAS40 and p70S6K). These changes were accompanied by robust suppression of AKT and associated signaling molecules (GSK3, PDK1), as well as loss of multiple transcription factors (NF-κB, STAT3, c-Myc, c-Jun, YAP, YB-1) that lie downstream of these signaling cascades. Increased expression of the cell cycle regulators p27 and Cyclin B1, along with loss of Cyclin E1, was seen and this finding is consistent with cell cycle arrest occurring as a consequence of ganetespib treatment. As expected we observed concomitant upregulation of HSP70 as well as caspase-7, a marker of apoptosis. Taken together, these coordinate impacts on multiple cellular pathways and processes conferred by Hsp90 blockade account for the potent activity of ganetespib in this IBC cell line.

**Table 2:** Fold-changes in protein expression following ganetespib treatment in SUM149 cells using reverse phase protein array analysis

Cellular target	Protein	Fold change
Receptor Tyrosine Kinases	IGF-1R beta	−1.8
	EGFR	−1.6
	EphA2	−1.5
AKT signaling	AKT (pS473)	−7.5
	GSK3 (S9)	−4.3
	GSK3-A/B (pS21/S9)	−3.9
	PDK1 (pS241)	−1.4
	AKT (pT308)	−1.4
	GSK3-A/B	−1.3
MAPK pathway	Src (pY527)	−2.6
	C-RAF (pS338)	−2.5
	JNK (pT183/Y185)	−1.7
	MAPK (pT202/Y204)	−1.6
	C-RAF	−1.5
	Src	−1.4
	Shc (pY317)	−1.3
Transcription factors	NF-κB p65 (pS536)	−3.3
	YAP	−2.2
	STAT3 (pY705)	−1.8
	c-Myc	−1.7
	c-Jun (pS73)	−1.5
	YB-1 (pS102)	−1.3
mTOR pathway	S6 (pS235/S236)	−6.5
	S6 (pS240/S244)	−6.2
	PRAS40 (pT246)	−2.3
	mTOR (pS2448)	−2.0
	4E-BP1 (pS65)	−1.9
	p70S6K	−1.4
	p70S6K (pT389)	−1.3
Cell cycle/arrest	Cyclin E1	−1.6
	p27	+1.3
	Cyclin B1	+1.8
Stress response	HSP70	+1.8
Apoptosis	Caspase-7 (cleaved D198)	+1.6
Other	Claudin-7	−1.5
	MIG-6	−1.4
	COX2	−1.3

Historically, single-modality treatments for IBC have not proven successful therefore combined agent strategies, often alongside surgery and radiation, represent the standard of care approach for treating IBC patients [41]. In light of these considerations, we investigated the combinatorial activity of ganetespib in combination with lapatinib, since HER2-targeted agents represent a novel and promising area of therapeutic intervention in IBC [42]. Concurrent administration of low (~ IC_40_) doses of ganetespib with lapatinib substantially increased cell death in SUM149 cells (Supplementary Fig. S1). The SUM149 line overexpresses EGFR and, as shown above, the EGFR/MAPK/STAT3 signaling axis was acutely sensitive to ganetespib treatment. Since lapatinib also inhibits EGFR activity, these data suggest that oncogenic signaling induced by this receptor represents a potential point of convergence between the two molecularly targeted agents.

## Discussion

Given the molecular complexity of breast cancer, it is now apparent that pharmacologic targeting of a single pathway or individual component of an oncogenic signal cascade typically fails to translate to long-term efficacy, particularly for metastatic disease. Due to a unique capacity to coordinately impact multiple signaling pathways in tumor cells, targeted inhibition of Hsp90 has emerged as a promising new strategy for cancer therapy [18]. For hormone-responsive (luminal-type) breast tumors, Hsp90 blockade represents a rational approach due to its well-defined role in the chaperoning of steroid receptors, including ER and PR [43]. With low nanomolar potency ganetespib destabilized both ER and PR in hormone-receptor positive breast cancer cells, leading to loss of viability and tumor growth suppression in xenograft models. Importantly, pharmacokinetic analysis confirmed that the loss of these client proteins was a durable response in vivo. Adjuvant endocrine therapy is the standard of care for patients with estrogen-dependent disease, and agents such as tamoxifen and aromatase inhibitors are effective first-line treatments [44]. However, there remains a need for alternative options in this population since the development of acquired resistance to these drugs is common, ultimately leading to relapse and patient death [45]. The mechanisms that underlie such refractory phenotypes remain poorly understood and, interestingly, tamoxifen resistance can arise even as the tumors themselves remain ER positive [46]. Previous reports have suggested that Hsp90 inhibition may overcome endocrine resistance in tamoxifen- and aromatase-resistant breast cancer cell lines [23, 47] and preliminary studies have shown that ganetespib exhibits such activity (D. Proia and L. Whitesell, unpublished data). Taken together, our data highlight the potential utility of ganetespib as an alternate, ligand-independent mechanism for sustained degradation of ER/PR in hormone receptor-positive breast tumors. In this regard, a randomized Phase II trial evaluating the activity of the estrogen receptor antagonist fulvestrant with or without ganetespib in hormone receptor-positive, metastatic breast cancer is underway (NCT 01560416).

Overexpression of HER2 defines a clinically relevant subset of breast tumors and the success of trastuzumab, a humanized monoclonal antibody targeting this oncoprotein, represents a significant landmark in the treatment of this disease [12]. HER2-positive breast cancer also provides a clear example of the relationship between client driver-protein dependence on Hsp90 and potential clinical efficacy [48], since this receptor is highly sensitive to Hsp90 chaperone inhibition and these tumors are exquisitely dependent on HER2-driven signaling for growth and survival. Consistent with reports for other Hsp90 inhibitors [24, 27, 28], ganetespib treatment was strongly cytotoxic to HER2-overexpressing cell lines in vitro and induced robust tumor regression in BT-474 xenografts. Notably, BT-474 cells retained sensitivity when grown as mammospheres and ganetespib also displayed superior potency to the dual HER2/EGFR tyrosine kinase inhibitor lapatinib in the three-dimensional culture system. Further, we showed that even brief exposure to ganetespib (as little as 5 min) resulted in a potent and sustained degradation of HER2 oncoprotein in this line. Overall this activity profile in HER2-driven tumor cells, a combination of potent oncogene inhibition with durable response properties, is likely to support the use of intermittent dosing schedules in the clinic.

Trastuzumab in combination with chemotherapy is the current standard of care for metastatic HER2-positive breast cancer [12]; however the invariable development of resistance remains a significant problem for this patient population. Recent clinical analyses have suggested a benefit for continued trastuzumab treatment beyond progression [49] and small molecule kinase inhibitors such as lapatinib have demonstrated efficacy in patients that become refractory to trastuzumab therapy [50]. Taken together these data indicate that trastuzumab-resistant tumors remain oncogenically reliant on HER2, suggesting that they may also remain sensitive to the effects of Hsp90 inhibition. A number of additional mechanisms have also been proposed to account for the resistant phenotype, including activation of compensatory growth factor signaling pathways, amplification of the PI3K/AKT cascade, and expression of truncated HER2 receptors that lack the antibody binding epitope (reviewed in [50]). Importantly, by virtue of its’ multifaceted mode of action, Hsp90 inhibition has been shown to overcome each of these various mechanisms in laboratory models of trastuzumab-resistant breast cancer [27, 51]. Therefore the use of Hsp90 inhibitors represents a rational approach to abrogate acquired resistance to primary anti-HER2 therapy. Clinical proof of concept has already been provided in a Phase II evaluation of the trastuzumab-tanespimycin combination in patients who had previously progressed on trastuzumab [29]. Despite the positive outcome of that trial, the continued development of tanespimycin as a cancer therapeutic has been discontinued by the sponsor. Ganetespib displays a superior activity, potency and safety profile over this first generation inhibitor [30] and thus represents a prime candidate for clinical evaluation, both as a single agent and combinatorial partner, in advanced HER2-positive breast cancer.

The activity profile of ganetespib presented here also highlights a new potential opportunity for therapeutic targeting in TNBC. Unlike HER2-positive or hormone receptor-positive breast cancer, TNBC represents a heterogeneous collection of orphan status tumors that lack a single defining vulnerability that serves as a druggable molecular target. As a result, a variety of potential biological drivers and molecular processes have been incompletely validated in this disease [8]. Among these, cell surface receptors such as EGFR, KIT and IGF-IR are established client proteins of Hsp90, as are central components of tumorigenic signaling cascades implicated in the pathogenesis of TNBC, including the RAS/RAF/ERK, PI3K/AKT and mTOR pathways [9]. Ganetespib displayed potent antitumor activity against TNBC cells, effectively and simultaneously degrading the EGFR, AKT, and mTOR signaling axes, leading to apoptosis and robust in vivo efficacy in xenograft models. These observations are in agreement with two recent reports that have provided similar preclinical evidence for the sensitivity of triple negative cancer cell lines to Hsp90 inhibition [25, 26].

Finally, we showed that ganetespib exposure caused pleiotropic effects on a variety of oncogenic pathways in IBC-derived SUM149 cells, including receptor tyrosine kinases, MAPK, AKT and mTOR signaling, transcription factors and proteins involved in cell cycle, stress and apoptotic regulation. IBC is not considered a histological subtype of breast cancer but rather a distinct clinicopathological entity—a rare yet highly aggressive type of locally advanced breast cancer with particularly poor prognosis [40]. Although IBC tumors may express any combination of hormone receptors and oncogenes they commonly exhibit *HER2* amplification, and EGFR overexpression is another characteristic associated with poor outcome [42]. IBC patients are typically treated by multi-modality approaches that integrate neoadjuvant chemotherapy, surgery and radiation therapy [41] and, if HER2 overexpression is present, trastuzumab-containing regimens have shown efficacy in the first-line setting [52, 53]. Overall, however, a molecular definition of IBC has not yet been developed and there are no established molecular criteria for distinguishing IBC from non-inflammatory type tumors [40]. Recently, amplification of anaplastic lymphoma kinase (ALK) was found with high frequency in IBC patient samples and cell lines, thereby identifying the first putative oncogenic driver for IBC [54]. If substantiated, these findings are not only biologically informative but provide a clinically-validated target for the development of new treatments for IBC patients. In this regard, it is important to note that ganetespib is a promising agent for ALK-driven tumors and can overcome multiple mechanisms of resistance to small molecule inhibitors of ALK currently in clinical practice [55].

In summary, our data show that targeting the Hsp90 chaperone complex with ganetespib represents a potentially effective strategy for therapeutic intervention across the broad spectrum of molecularly-defined, histological subtypes of breast cancer. The compound potently and simultaneously inhibits multiple oncogenic signaling pathways in breast cancer cell lines in vitro and potentiates the activity of other standard of care and molecularly targeted agents presently used in the management of breast cancer. Ganetespib also displays robust in vivo activity over a variety of dosing regimens covering daily to weekly schedules, potentially providing a high degree of flexibility in dose and schedule within the clinical setting. Overall, these findings provide a strong rationale to support the evaluation of ganetespib as a new treatment option for breast cancer patients. Accordingly, ganetespib has currently entered Phase II evaluation as a front-line therapy for HER2-positive and triple negative metastatic breast cancers.

## Electronic supplementary material

Below is the link to the electronic supplementary material.

Fig. S1 (JPEG 20 kb)

High Resolution Image (TIFF 214 kb)
