# Supplementary figures and images for: Targeted inhibition of Hsp90 by ganetespib is effective across a broad spectrum of breast cancer subtypes
Source: Invest New Drugs. 2013 May 18;32(1):14–24. doi: 10.1007/s10637-013-9971-6 (PMC3913847; doi:10.1007/s10637-013-9971-6)

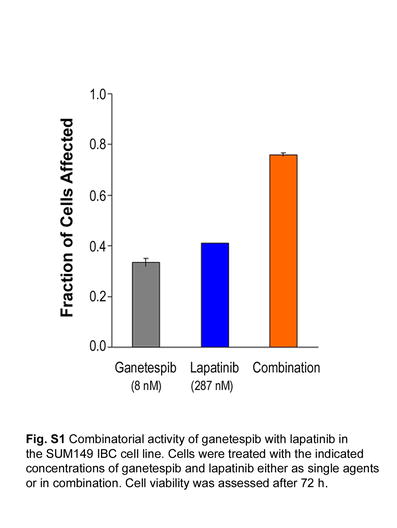

Supplement: Supplementary file 1 — (JPEG 20 kb) [file 10637_2013_9971_Fig5_ESM.jpg]

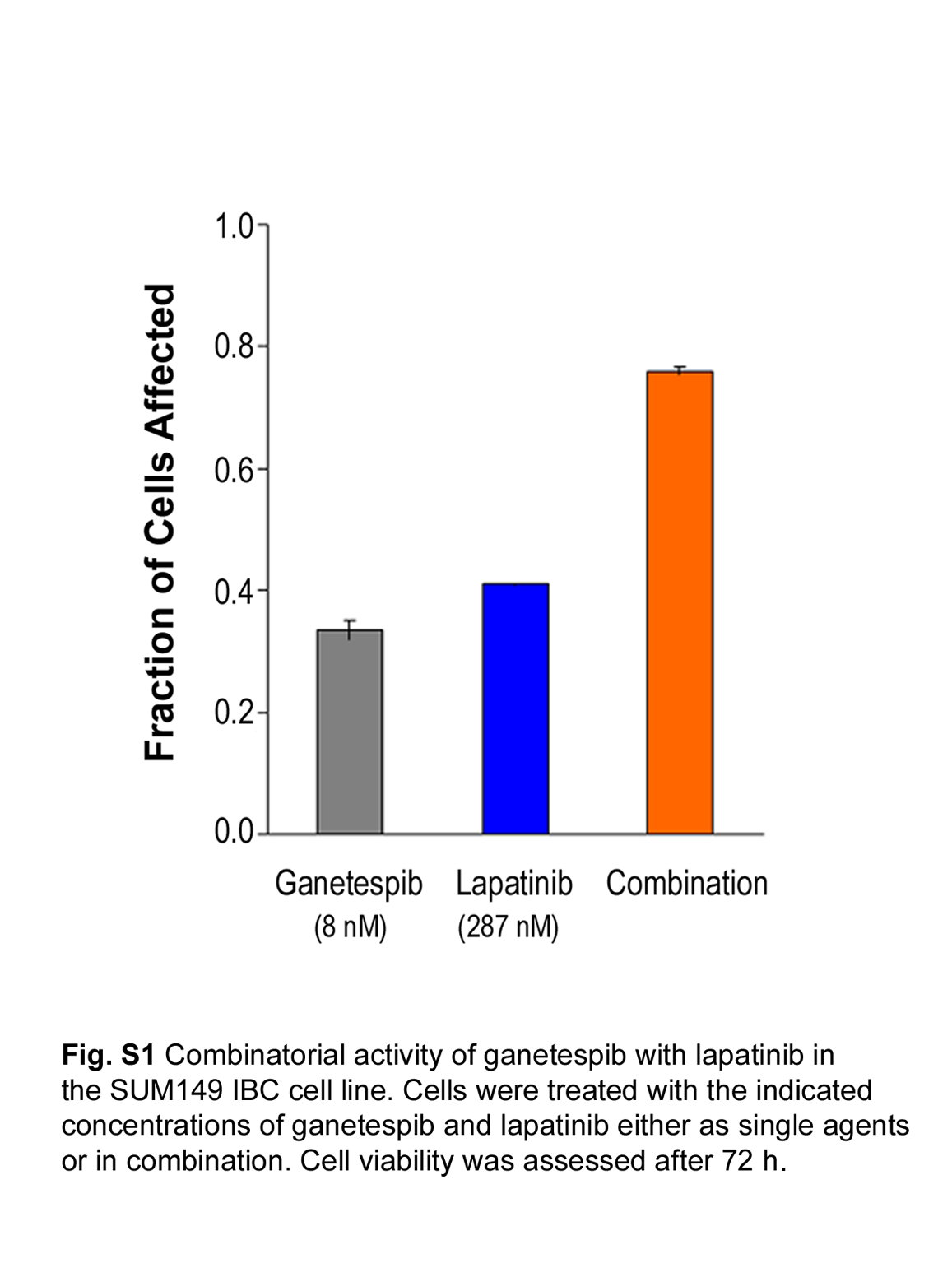

Supplement: Supplementary file 2 — (TIFF 214 kb) [file 10637_2013_9971_MOESM1_ESM.tif]
